# Supplementary material for: Development of a Model for Genomic Prediction of Multiple Traits in Common Bean Germplasm, Based on Population Structure
Source: Plants (Basel). 2022 May 12;11(10):1298. doi: 10.3390/plants11101298 (PMC9144439; doi:10.3390/plants11101298)
Supplement: Supplementary file 1 [file plants-11-01298-s001.zip › SFigure_CommonBean_new.pdf]

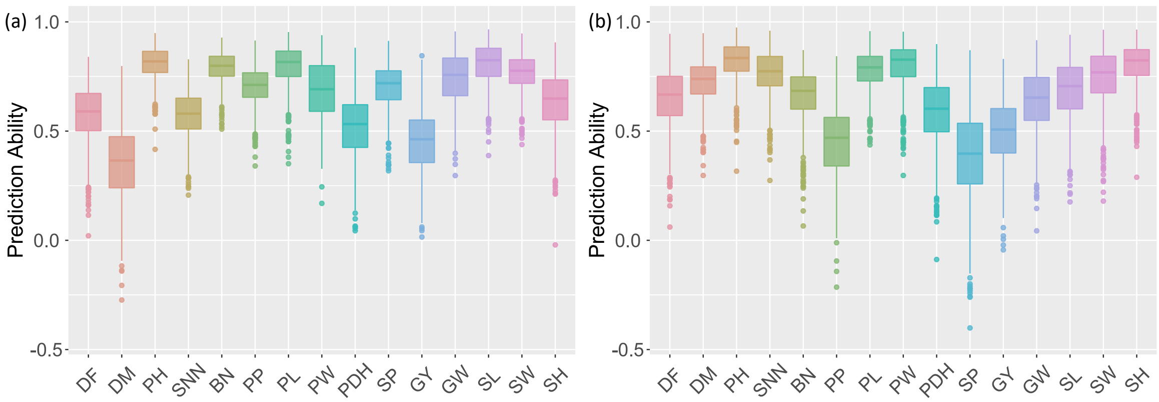

Supplementary Figure S1. Ten-fold cross validation with 100 replications were performed for 15 agronomic traits in landraces from two common bean gene pools, respectively. (a) Andean gene pool landraces (223); (b) Mesoamerican gene pool landraces (261).

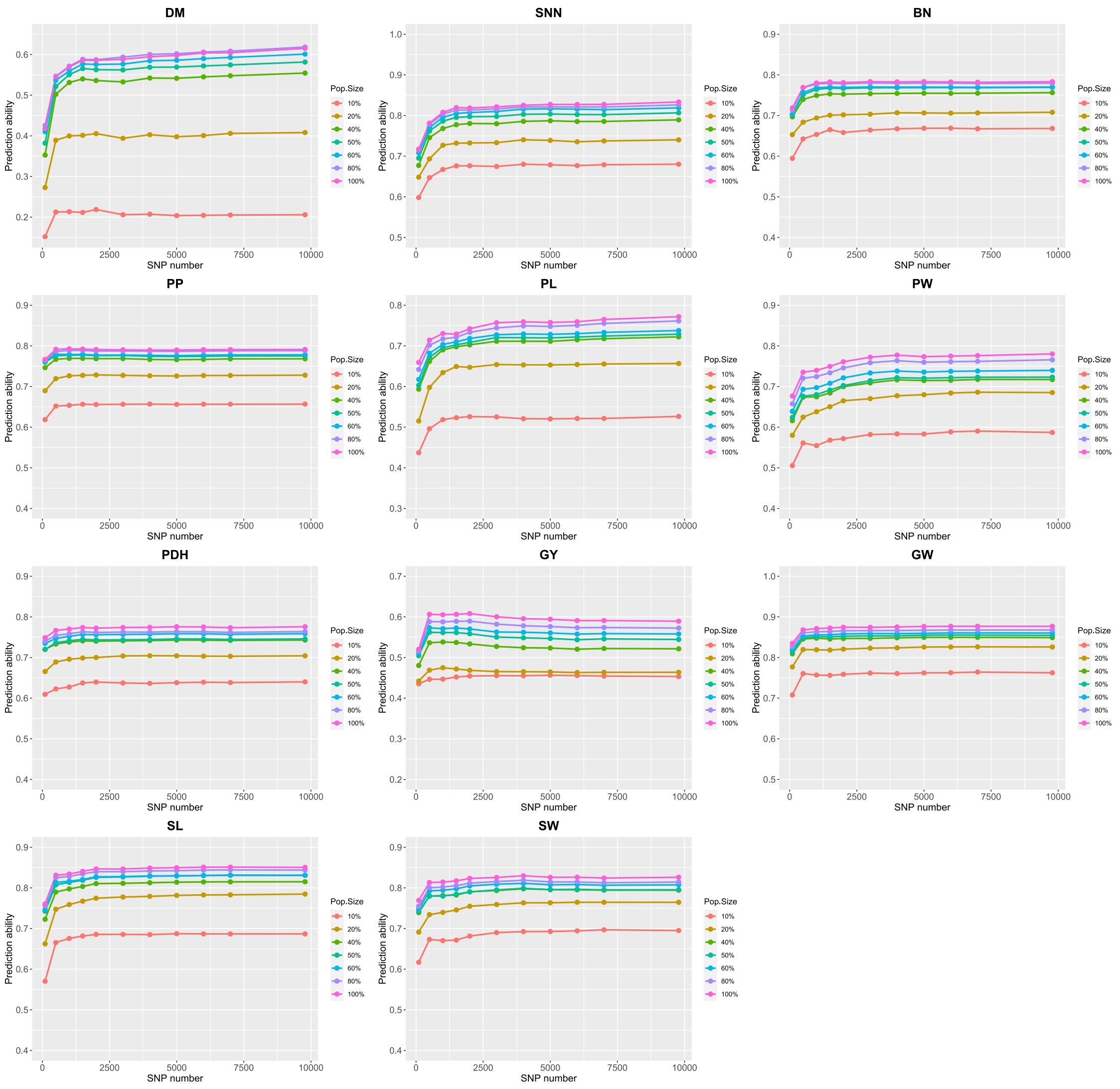

Supplementary Figure S2. Different number of SNPs (100, 500, 1000, 1500, 2000, 3000, 4000, 5000, 6000, 7000 and 9781) and different population size (10, 20, 40, 50, 60, 80 and 100%) were used in ten-fold cross validation with 100 replications for 11 agronomic traits in 484 common bean landraces.

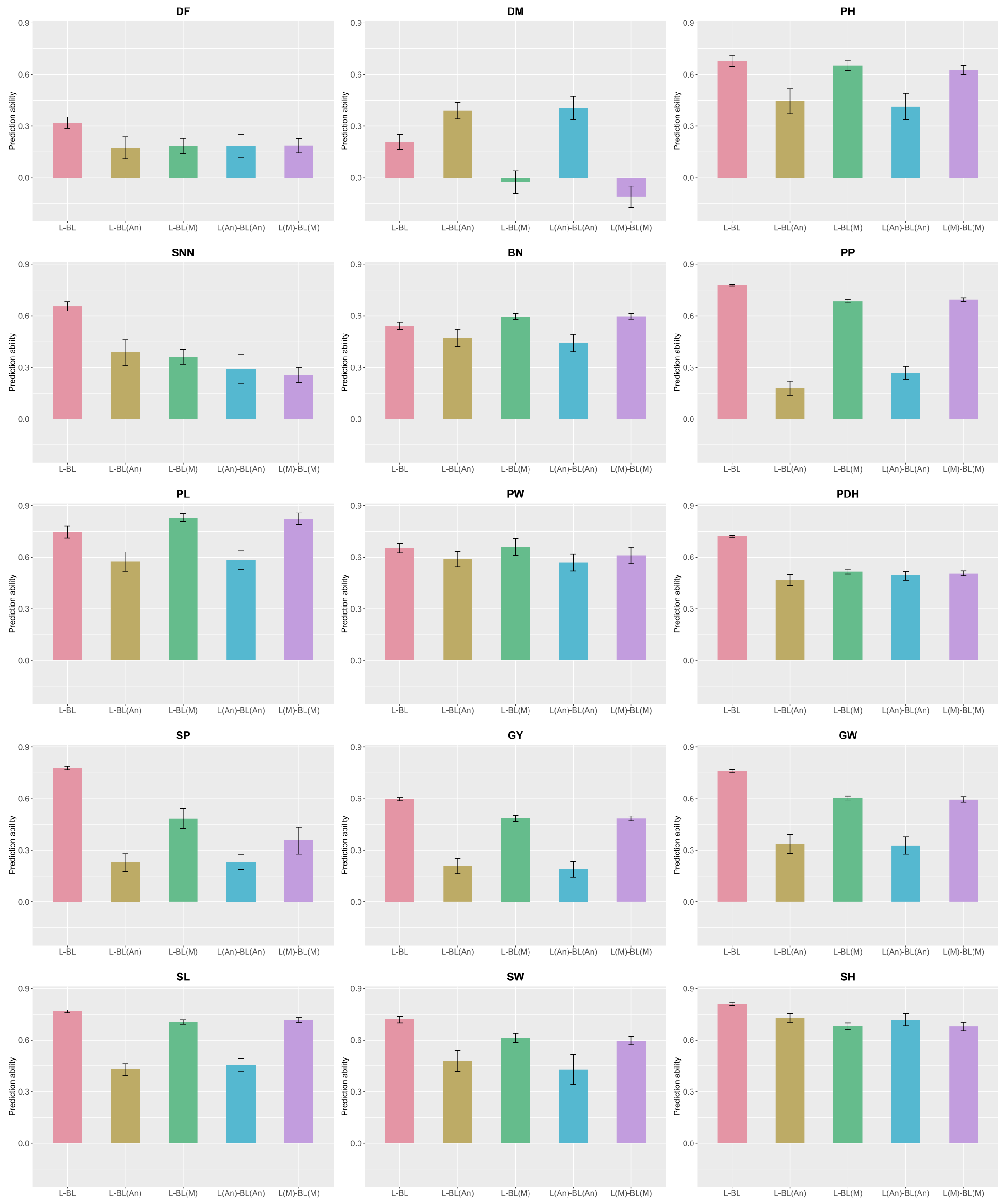

Supplementary Figure S3. Histograms of prediction ability of landraces as training set to predict breeding lines among 15 traits. (i.e., The abscissa represents the training set-testing set.)

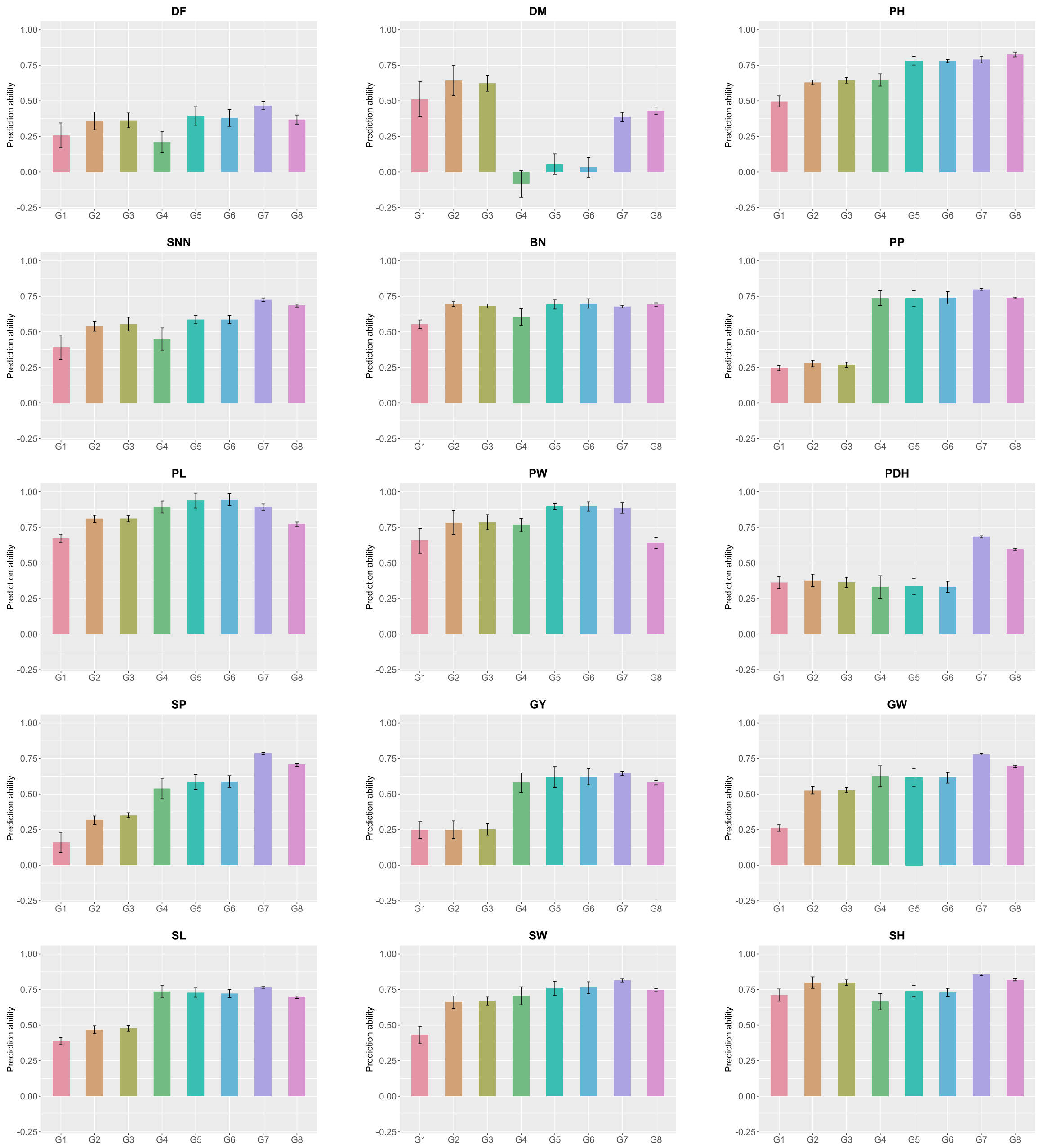

Supplementary Figure S4. Histograms of model's precision for 8 Groups of two scenarios among 15 traits. (G1, G2 and G3: Andean gene pool; G4, G5 and G6: Mesoamerican gene pool; G7 and G8: Entire gene pool.)
