# Supplementary material for: Development of a Model for Genomic Prediction of Multiple Traits in Common Bean Germplasm, Based on Population Structure
Source: Plants (Basel). 2022 May 12;11(10):1298. doi: 10.3390/plants11101298 (PMC9144439; doi:10.3390/plants11101298)
Supplement: Supplementary file 1 [file plants-11-01298-s001.zip › STable_CommonBean_new.pdf]

Supplementary Table S1. Abbreviations and description for fifteen traits.

Traits	Abbreviations	Description
days to flowering	DF	Days from sowing to flowering
days to maturity	DM	Days from sowing to maturity
plant height	PH	At maturity, the distance from the cotyledon node of the main stem to the top of the plant
stem node number	SNN	At maturity, the number of nodes from the cotyledons of the main stem to the top of the plant
branch number	BN	Number of primary branches on main stem
pod per plant	PP	At maturity , number of mature pods per plant
pod length	PL	At maturity, measure the straight-line distance from the pod tip to the pod tail of the dry ripe pod
pod width	PW	At maturity, measure the straight line distance from the widest part of the dry ripe pod
pod height	PDH	At maturity, the distance between the upper and lower pod surfaces
seeds per pod	SP	At maturity, the number of mature kernels contained in the dry ripe pods
grain yield per plant	GY	At maturity, dry kernel weight per plant
100-grain weight	GW	Weight of 100 ripe dry kernels
seed length	SL	Parallel to the navel, the distance between the top and bottom vertices
seed width	SW	Perpendicular to the navel, the distance between the upper and lower surfaces
seed height	SH	The distance from the navel to its opposite

Phenotypes were taken from the published, Wu et al., 2020.

Supplementary Table S2. Mean values of prediction abilities for 15 agronomic traits in the landraces from two common bean gene pools, respectively.

Traits	CV(10*100)	
	An(223)	M(261)
DF	0.6494 (0.1331)	0.5769 (0.1285)
DM	0.7252 (0.0944)	0.3540 (0.1733)
PH	0.8213 (0.0868)	0.8105 (0.0727)
SNN	0.7655 (0.1004)	0.5759 (0.1031)
BN	0.6649 (0.1164)	0.7934 (0.0677)
PP	0.4466 (0.1609)	0.7054 (0.0860)
PL	0.7796 (0.0856)	0.7991 (0.0939)
PW	0.8037 (0.0974)	0.6868 (0.1351)
PDH	0.5900 (0.1458)	0.5173 (0.1417)
SP	0.3872 (0.2046)	0.7041 (0.1034)
GY	0.4979 (0.1477)	0.4514 (0.1357)
GW	0.6391 (0.1420)	0.7407 (0.1185)
SL	0.6876 (0.1379)	0.8094 (0.0940)
SW	0.7495 (0.1226)	0.7665 (0.0828)
SH	0.8024 (0.0953)	0.6358 (0.1312)

The standard deviations of the prediction abilities were presented in brackets.

Supplementary Table S3. The model's precision of 484 landraces using different numbers of SNPs and different population size for 15 traits. (The prediction ability results in **Figure 2**.)

Traits	Broad sense heritability			All SNPs and populations (PA)	opt_snp	opt_pops	Optimal SNPs and population (PA)
	BIJ	HA	HN				
**Days to Flowering**	0.9245	0.9557	0.8132	0.7266	2k	40%	0.6732
**Days to Maturity**	0.8214	0.9162	0.7735	0.6152	1.5k	50%	0.5656
**Plant Height**	0.9323	0.9616	0.9434	0.8823	1k	40%	0.8436
**Stem Node Number**	0.8510	0.6121	0.9083	0.8332	1.5k	50%	0.7953
**Branch Number**	0.6633	0.4658	0.6664	0.7832	1k	50%	0.7644
**Pods per Plant**	0.7154	0.7152	0.7282	0.7911	0.5k	40%	0.7670
**Pod Length**	0.9023	0.9048	0.9517	0.7719	2k	40%	0.7025
**Pod Width**	0.8881	0.6332	0.9257	0.7802	2k	40%	0.6997
**Pod Height**	NA	NA	NA	0.7758	0.5k	40%	0.7331
**Seeds per Pod**	0.8387	0.8650	0.8911	0.8249	0.5k	40%	0.7738
**Grain Yield per Plant**	0.6209	0.5721	0.5174	0.5893	0.5k	50%	0.5622
**100-grain Weight**	0.9549	0.9374	0.9709	0.8768	0.5k	40%	0.8456
**Seed Length**	0.9733	0.9586	0.9660	0.8505	0.5k	40%	0.7898
**Seed Width**	0.9469	0.8975	0.9361	0.8261	0.5k	40%	0.7798
**Seed Height**	0.8991	0.9223	0.9385	0.8352	0.5k	40%	0.8143

Broad sense heritability is taken from the published Wu et al., 2020.

Supplementary Table S4. The table records the number of identical materials in the material corresponding to the top 30 (as well as the top 30%) predicted and observed values in the testing set. (i.e., the numerator of the ratio in the table)

Traits	Training set-Testing set (Size of breeding lines)		Top 30	Top 30%	Traits	Training set-Testing set (Size of breeding lines)		Top 30	Top 30%
**DF**	L-BL	(144)	10/30	18/43	**PP**	L-BL	(144)	18/30	32/43
	L-BL(An)	(60)	17/30	5/18		L-BL(An)	(60)	16/30	9/18
	L-BL(M)	(84)	12/30	9/25		L-BL(M)	(84)	17/30	14/25
	L(An)-BL(An)	(60)	18/30	4/18		L(An)-BL(An)	(60)	19/30	9/18
	L(M)-BL(M)	(84)	12/30	9/25		L(M)-BL(M)	(84)	18/30	15/25
**DM**	L-BL	(144)	8/30	13/43	**PL**	L-BL	(144)	16/30	28/43
	L-BL(An)	(60)	17/30	7/18		L-BL(An)	(60)	22/30	8/18
	L-BL(M)	(84)	9/30	7/25		L-BL(M)	(84)	20/30	19/25
	L(An)-BL(An)	(60)	20/30	9/18		L(An)-BL(An)	(60)	21/30	8/18
	L(M)-BL(M)	(84)	10/30	7/25		L(M)-BL(M)	(84)	22/30	20/25
**PH**	L-BL	(144)	18/30	24/43	**PW**	L-BL	(144)	19/30	32/43
	L-BL(An)	(60)	18/30	9/18		L-BL(An)	(60)	23/30	10/18
	L-BL(M)	(84)	18/30	16/25		L-BL(M)	(84)	20/30	16/25
	L(An)-BL(An)	(60)	19/30	9/18		L(An)-BL(An)	(60)	24/30	10/18
	L(M)-BL(M)	(84)	18/30	14/25		L(M)-BL(M)	(84)	21/30	16/25
**SNN**	L-BL	(144)	15/30	22/43	**PDH**	L-BL	(144)	19/30	32/43
	L-BL(An)	(60)	16/30	10/18		L-BL(An)	(60)	20/30	12/18
	L-BL(M)	(84)	14/30	12/25		L-BL(M)	(84)	22/30	15/25
	L(An)-BL(An)	(60)	13/30	11/18		L(An)-BL(An)	(60)	21/30	12/18
	L(M)-BL(M)	(84)	15/30	12/25		L(M)-BL(M)	(84)	21/30	16/25
**BN**	L-BL	(144)	7/30	11/43	**SP**	L-BL	(144)	13/30	29/43
	L-BL(An)	(60)	20/30	6/18		L-BL(An)	(60)	19/30	9/18
	L-BL(M)	(84)	13/30	10/25		L-BL(M)	(84)	14/30	10/25
	L(An)-BL(An)	(60)	21/30	6/18		L(An)-BL(An)	(60)	19/30	9/18
	L(M)-BL(M)	(84)	14/30	10/25		L(M)-BL(M)	(84)	17/30	13/25

Traits	Training set-Testing set (Size of breeding lines)		Top 30	Top 30%
**GY**	L-BL	(144)	14/30	29/43
	L-BL(An)	(60)	18/30	5/18
	L-BL(M)	(84)	17/30	12/25
	L(An)-BL(An)	(60)	18/30	5/18
	L(M)-BL(M)	(84)	16/30	12/25
**GW**	L-BL	(144)	19/30	29/43
	L-BL(An)	(60)	22/30	8/18
	L-BL(M)	(84)	24/30	19/25
	L(An)-BL(An)	(60)	20/30	6/18
	L(M)-BL(M)	(84)	25/30	19/25
**SL**	L-BL	(144)	15/30	29/43
	L-BL(An)	(60)	22/30	10/18
	L-BL(M)	(84)	24/30	18/25
	L(An)-BL(An)	(60)	23/30	11/18
	L(M)-BL(M)	(84)	25/30	18/25
**SW**	L-BL	(144)	18/30	29/43
	L-BL(An)	(60)	20/30	12/18
	L-BL(M)	(84)	19/30	16/25
	L(An)-BL(An)	(60)	21/30	10/18
	L(M)-BL(M)	(84)	20/30	14/25
**SH**	L-BL	(144)	16/30	30/43
	L-BL(An)	(60)	21/30	10/18
	L-BL(M)	(84)	21/30	17/25
	L(An)-BL(An)	(60)	20/30	10/18
	L(M)-BL(M)	(84)	21/30	18/25
